# NuXLApp: Interactive data analysis and visualization for protein‐nucleic acid crosslinking mass spectrometry

**DOI:** 10.1002/pro.70820

**Published:** 2026-10-15

**Authors:** Arslan Siraj, Sergei Moshkovskii, Tom David Müller, Henning Urlaub, Oliver Kohlbacher, Timo Sachsenberg

**Affiliations:** ^1^ Applied Bioinformatics, Department of Computer Science University of Tübingen Tübingen Germany; ^2^ Institute for Bioinformatics and Medical Informatics University of Tübingen Tübingen Germany; ^3^ Department of Clinical Chemistry University Medical Center Göttingen Göttingen Germany; ^4^ Research Group Bioanalytical Mass Spectrometry Max Planck Institute for Multidisciplinary Sciences Göttingen Germany; ^5^ Translational Bioinformatics University Hospital Tübingen Tübingen Germany

**Keywords:** computer science, data processing, false discovery rate, mass spectrometry, proteomics, raw data, search engine, visualization, web application, web application framework

## Abstract

Protein‐nucleic acid (protein‐Nu) interactions are crucial for many cellular processes and biological functions. Crosslinking mass spectrometry (XL‐MS) has emerged as a powerful method for identifying these interactions. However, the analysis of protein‐Nu XL‐MS data requires an integrated workflow from raw data processing to results inspection and validation. Existing workflows for protein‐Nu XL‐MS often require switching between tools and considerable manual processing. Here, we introduce NuXLApp, an interactive web‐based application for data processing and results visualization in protein‐Nu XL‐MS studies. NuXLApp integrates the protein‐Nu crosslink search engine “NuXL,” machine‐learning models for peptide property prediction, and interactive result visualization within a reproducible workflow. It enables rapid validation of false discovery rate‐controlled crosslink spectrum match‐level identifications and facilitates collaboration and intuitive exploration of experimental results. In addition, NuXLApp generates a spectral library from identified protein‐Nu crosslinks to enable quantitative analysis of crosslinks using data‐independent acquisition (DIA). NuXLApp supports discovery‐driven identification of protein‐nucleic acid crosslinks and enables DIA‐based follow‐up experiments for their quantitative analysis. NuXLApp is open‐source and available via the OpenMS WebApps platform at https://nuxl.webapps.openms.de/.

## 1 INTRODUCTION

From the earliest stages of life, interactions between nucleic acids and proteins have been fundamental to biological systems (Altstein, 2015). Biomolecular complexes involving DNA or RNA and proteins are essential for major cellular processes, including DNA replication, transcription, chromatin remodeling, RNA splicing, and translation (Cozzolino et al., 2021; Mitsis et al., 2020; Staley & Woolford Jr., 2009). Disruptions in these interactions are associated with inherited and acquired disorders, such as diabetes, cancer, and neurodegenerative and cardiovascular diseases (Gebauer et al., 2021; Hemphill et al., 2023). Emerging high‐throughput approaches, such as UV light‐mediated or chemical crosslinking followed by high‐resolution liquid chromatography‐mass spectrometry (LC–MS), allow these interactions to be studied not only in isolated macromolecular complexes but also in entire cellular systems (Welp et al., 2025).

Currently, mass spectrometry using data‐dependent acquisition (DDA) is employed to identify peptide crosslinks carrying one to three nucleotides across the proteome (Kramer et al., 2014; Stützer et al., 2020; Trendel et al., 2019; Trendel et al., 2025; Welp et al., 2025). The site localization of these crosslinks can be determined with accuracy to a single amino acid residue, enabling proteome‐wide mapping of nucleic‐acid‐binding regions in proteins (Welp et al., 2025). In addition, quantitative analysis of these crosslinks by DIA is a promising next step, as it could enable comparative analysis of protein‐RNA interactomes across different biological states.

Several computational tools have been developed for the identification and analysis of protein‐Nu crosslinks from LC–MS data. RNPxl introduced an automated workflow for the analysis of UV‐induced protein‐RNA crosslinking experiments and enabled the identification of peptide‐RNA heteroconjugates from high‐resolution MS/MS spectra (Kramer et al., 2014). RNxQuest extended the xQuest pipeline to protein‐RNA XL‐MS data and provided a search strategy particularly suited to isotope‐labeled protein‐RNA crosslinks (Sarnowski et al., 2023). MSFragger‐Labile introduced a general labile‐modification search strategy that can improve the identification of labile modifications, including protein‐RNA crosslinked peptides (Polasky et al., 2023). Among these approaches, NuXL provides a dedicated search strategy for protein‐Nu XL‐MS and was specifically developed for the analysis of crosslinked protein‐Nu samples (Welp et al., 2025). NuXL supports different crosslink modification chemistries, including UV‐induced and novel chemically induced crosslinking protocols, making it well suited for the analysis of diverse protein‐Nu XL‐MS datasets. While NuXL provides a dedicated search engine for protein‐Nu XL‐MS, its routine application still requires several separate software components for data processing, downstream analysis, and visualization. NuXL result visualization is often performed using TOPPView (Sturm & Kohlbacher, 2009), where users must load both the mzML file and the corresponding identification file to view annotated spectra. To utilize spectrum inspection capabilities in TOPPView, users must switch between tools and manage multiple files. To analyze samples with the NuXL search engine, users must install OpenMS as a desktop application or build it from source (Pfeuffer et al., 2024). For downstream analysis (e.g., rescoring with data‐driven features), users need to create a Python environment to install the necessary packages (Siraj et al., 2024). Users thus spend considerable time performing manual tasks, such as transferring data between multiple software platforms. These manual interventions are not only time‐ and resource‐intensive but also introduce variability, decrease reproducibility, and pose challenges to achieving consistent and reliable scientific outcomes. An integrated workflow platform that seamlessly combines data processing and visualization can help address these challenges.

Protein‐Nu XL‐MS data analysis benefits from software that integrates processing and visualization in a single environment. Here we present NuXLApp, an open‐source, platform‐independent web application for interactive analysis and visualization of protein‐Nu XL‐MS samples.

## 2 METHODS

### 2.1 NuXLApp implementation and architecture

NuXLApp (version 2.0.0) is implemented using the OpenMS web application template and developed as a Streamlit (version 1.38.0) web application (Müller et al., 2025). NuXLApp is built with Python (version 3.10.0) and leverages the OpenMS NuXL search engine (built on the develop branch and tagged NuXLApp). The NuXLApp interface is organized into modular Streamlit pages for Quick Start, user documentation, input file management, NuXL search, rescoring, DIA spectral library generation and result dashboard (Figure 1). Navigation is provided via a sidebar, which also allows users to configure export settings for figures and tables. Upon launching NuXLApp, users are directed to the Quick Start page, which introduces the application's functionality and provides an overview of the implemented analysis workflows. The Documentation page provides step‐by‐step guidance for performing the available analyses. Three dedicated workflow pages implement the NuXL search, rescoring with data‐driven features, and DIA spectral‐library generation. The Results page enables interactive inspection and export of newly generated results and previously completed NuXL analyses.

**FIGURE 1:**
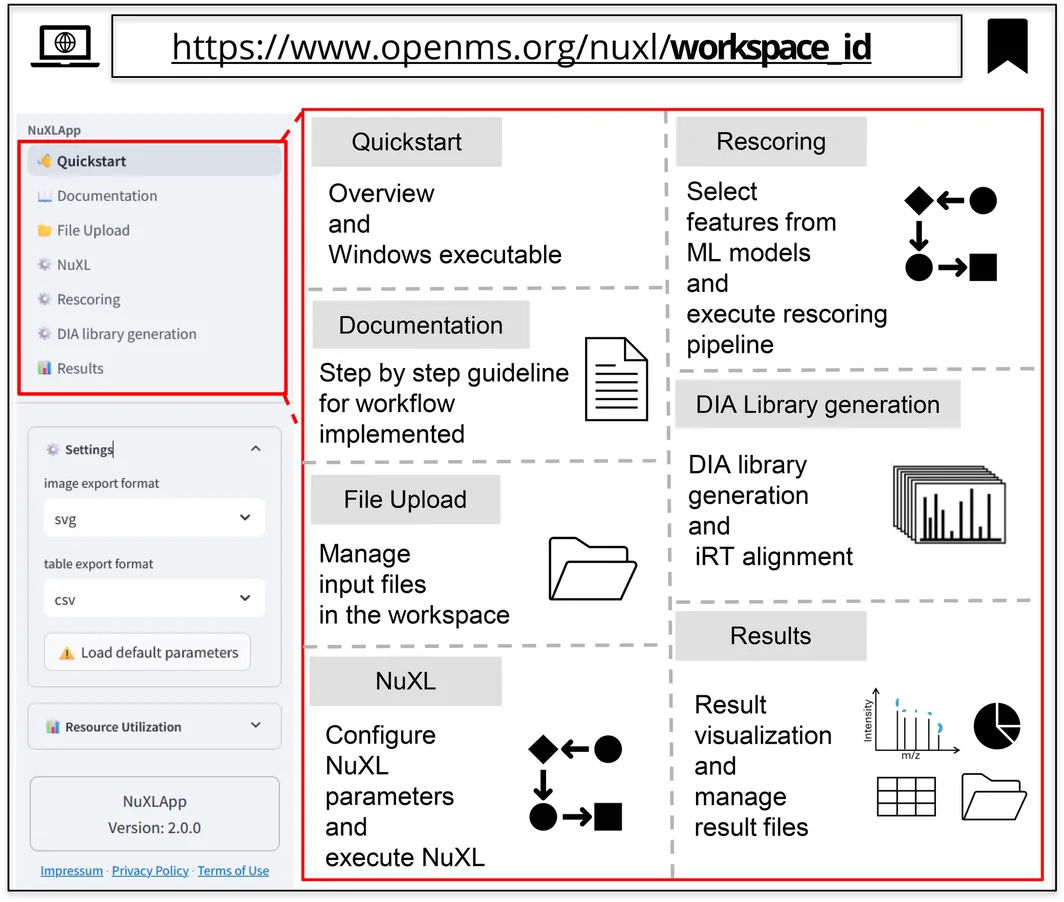
Overview of the NuXLApp interface. The sidebar facilitates navigation through NuXLApp, providing access to the main workflow steps (File Upload, run NuXL, Rescoring, DIA library generation, and Results) and settings for export formats. The workspace ID is encoded in the URL. The Quick Start page provides an overview of NuXLApp and a downloadable Windows executable. The Documentation page offers step‐by‐step guidance for using the implemented analysis workflows. The File Upload page allows users to upload and manage NuXL input files (.mzML, .raw, and .fasta). The NuXL page enables users to configure and execute the NuXL tool. The Rescoring page allows users to configure and execute the rescoring pipeline. The DIA library generation page allows users to generate a DIA library with optional iRT alignment. All analysis workflows provide real‐time logging. The Results page displays the results dashboard, which provides interactive visualizations, including a CSM table with annotated spectra, crosslink efficiency, and a summary of crosslink modifications (see Figures S4–S7). Users can manage the result files and upload the NuXL output files to quickly visualize already analyzed data.

NuXLApp is available in multiple deployment modes to accommodate diverse usage scenarios. It can be accessed as an online service using Docker‐based containerization or executed locally via a standalone Windows executable. The Windows installer (.msi) is available through the NuXLApp interface or the GitHub releases page.

### 2.2 Workspaces and reproducibility

NuXLApp employs a workspace‐based architecture to support reproducible analyses. The workflow diagram (Figure 2) shows the end‐to‐end analysis within NuXLApp, from data upload and NuXL search to rescoring, DIA spectral library generation, and interactive result interpretation, all managed within a dedicated workspace to support reproducible analysis. Each computational workflow follows a standardized procedure comprising file specification, parameter configuration, and workflow execution (see Figures S1–S3, Supporting Information). This structure enables users to verify the selected input files and analysis settings before execution and to monitor workflow progress through live logging. Input data, parameter configurations, execution records, and generated results remain associated with the workspace, while outputs from one workflow stage are directly available for subsequent analysis steps and result visualization. When NuXLApp is accessed online, a new workspace is instantiated automatically at session start and its identifier is encoded in the application URL, allowing analyses to be bookmarked and revisited. The URL can be shared, enabling collaborative access to the same workspace among multiple users. In local mode, users can manually create and manage workspaces, providing full control over the organization of data across multiple projects.

**FIGURE 2:**
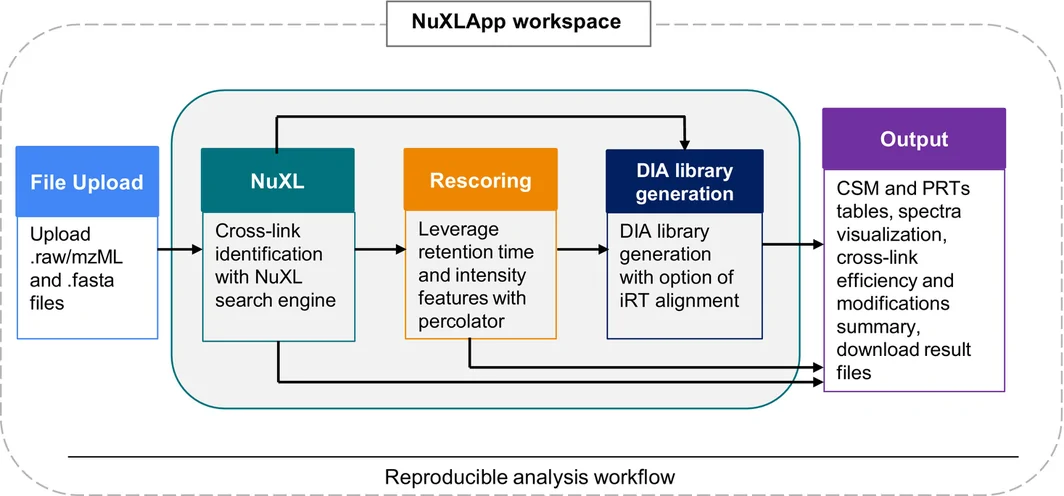
End‐to‐end NuXLApp workflow for protein‐nucleic acid XL‐MS data analysis. Thermo Raw or mzML mass spectrometry data and corresponding FASTA files are uploaded to the NuXLApp workspace and processed with the NuXL search engine for crosslink identification. The resulting identifications can be inspected directly or, optionally, subjected to Percolator‐based rescoring using retention‐time and fragment‐intensity features. Original or rescored identifications can subsequently be used to generate a DIA‐NN‐compatible spectral library with optional iRT alignment. All workflow modules operate within the same workspace to support a reproducible analysis process. The outputs include CSM and PRT tables, annotated crosslinked spectra, summaries of crosslinking efficiency and precursor modifications, and downloadable result files.

### 2.3 File upload and NuXL analysis

Input data are provided through the File Upload page, where users import mass spectrometry data in either Thermo (.raw) or mzML format, together with the corresponding FASTA sequence database. NuXL parameters are configured on the NuXL page, which provides standard and advanced settings for the NuXL search engine (Figure S1). During execution, real‐time logs are displayed to allow users to monitor progress, and running analyses can be terminated at any time. After successful completion of the NuXL search engine run, the output files can be downloaded and subsequently used for rescoring, DIA library generation, and visualization.

### 2.4 Rescoring

NuXLApp integrates the NuXL‐rescore pipeline, which is accessible through the Rescoring page (Figure S2) (Siraj et al., 2024). For rescoring, users can directly use the output generated by the NuXL search engine; the NuXL output file produced prior to false discovery rate (FDR) calculation is required as input for the rescoring pipeline. Machine learning derived features, including predicted retention times and fragment ion intensities, are computed and combined with the original NuXL search engine features and supplied to Percolator for rescoring, as described in our rescoring study (Siraj et al., 2024). Retention time‐based features are calculated following the approach used in MS2Rescore (Declercq et al., 2022), using predictions from a DeepLC model fine‐tuned on protein‐Nu crosslinks. Fragment ion intensities are predicted using the MS2PIP HCD XGBoost‐based machine learning model trained on tryptic and non‐tryptic (immunopeptide) HCD peptides. Intensity‐based features are computed as the maximum Pearson correlation between predicted and observed intensities of the first three prefix and suffix fragment ions. Calculation of these intensity‐based features requires an MS file (.mgf). If a corresponding MGF file is not already available, NuXLApp automatically converts the matching MS file (.mzML) during rescoring. After successful completion of the rescoring analysis, a Pseudo‐ROC plot is generated for comparison, and the output files are copied to the workspace for subsequent DIA library generation and visualization. To clearly distinguish rescored results from the original NuXL output files, all files generated by the rescoring pipeline are labeled with the prefix RDDF_ (Rescoring with Data‐Driven Features).

### 2.5 Spectral library generation

NuXLApp integrates a Python‐based converter that transforms NuXL DDA search results into DIA‐NN‐compatible spectral libraries (Figure S3) (Demichev et al., 2020). A spectral library can be generated either directly from NuXL output files or from rescored crosslink spectrum match (CSM) identifications, allowing users to construct libraries from identifications with improved confidence after data‐driven rescoring. As input, the converter uses identification files (.idXML) containing crosslinked identifications filtered at 1% CSM‐level FDR and linear peptide‐spectrum matches (PSMs) filtered at 1% PSM‐level FDR. The CSM‐ and PSM‐level FDRs are estimated separately from the corresponding target and decoy identifications. Specifically, the CSM‐level FDR is estimated from target and decoy CSMs, whereas the PSM‐level FDR is estimated from target and decoy linear PSMs. Decoy sequences are generated automatically by the NuXL search engine during the target‐decoy database search. Following in‐silico digestion according to the selected enzyme specificity, peptide sequences are deterministically shuffled between cleavage sites while retaining the C‐terminal residue. Up to 100 shuffling attempts are performed, and a shuffled sequence is selected if it does not share prefixes or suffixes with the corresponding target peptide; otherwise, the sequence with the lowest similarity to the target peptide is retained (Welp et al., 2025). Decoy identifications are recognized by the “DECOY_” prefix in the protein accession. If decoy identifications are present, they are removed before spectral library construction. Crosslinked identifications are further filtered using a localization score threshold of >0.0. Redundant precursors are removed by retaining the best‐scoring representative per sequence, nucleotide adduct, and charge state for crosslinks, or per sequence and charge state for linear peptides. CCS values, when available, are converted to reduced ion mobility (1/K0) using the Mason‐Schamp equation. Modified peptide sequences are reformatted according to the DIA‐NN convention, and nucleotide adduct annotations are assigned to the position reported by the NuXL localization. Fragment annotations are parsed from the NuXL output file, and only b‐ and y‐type fragment ions are retained. Optional retention time normalization to iRT can be performed using an external FragPipe spectral library generated from quality‐control samples measured under comparable LC conditions, such as HeLa lysates (Yu et al., 2023). NuXL retention time values are converted to iRT values using the FragPipe output and piecewise or linear calibration. The resulting spectral library is exported in tab‐separated file format.

### 2.6 Result visualization and interpretation

The Results page provides integrated visualizations and tabular outputs to support comprehensive interpretation of NuXL analysis results. In addition to displaying newly generated outputs, the interface allows users to upload already analyzed result files, enabling rapid validation and comparison of results. NuXL output files are named according to the CSM‐level FDR threshold applied during filtering, with the numeric suffix indicating the FDR cutoff, where 0.0100, 0.1000, and 1.0000 correspond to 1%, 10%, and 100% CSM‐level FDR, respectively; the 1.0000 files therefore contain the unfiltered identifications. Identified CSMs are presented in an interactive CSM table (Figure S4); selection of an individual CSM dynamically generates the corresponding ion‐annotated MS/MS spectrum, in which fragment ions are visualized using a distinct color scheme for improved interpretability. For individual CSMs, users can restrict the displayed annotations to selected ion types, including b ions, y ions, combined b/y ions, marker ions (MI), and precursor ions, while retaining the complete experimental spectrum when the corresponding mzML file is available. Protein‐level summaries of crosslinked sites are provided through the proteins (PRTs) table (Figure S5), allowing assessment of the distribution of crosslink identifications across proteins, including unique and shared peptide assignments. Crosslinking preferences are further characterized by a crosslink efficiency analysis, displayed as a bar plot showing the relative frequency of crosslinked amino acids normalized to their overall occurrence across all CSMs (Figure S6). This analysis helps identify amino acids that are preferentially involved in crosslinking within the sample. The distribution of precursor adducts is summarized in a pie chart, facilitating identification of the most prevalent crosslink modifications present in the experiment (Figure S7). All tables and plots are interactive and can be downloaded in user‐selected formats, including CSV and TSV for tabular results and SVG or PNG for graphical outputs.

### 2.7 Reproducible workflow execution

The benchmark analyses presented in this work were conducted using example input files that are directly accessible within NuXLApp, ensuring full reproducibility using identical data and parameter settings. To evaluate the performance of the rescoring pipeline on published data, a single raw data file generated using the protein‐RNA DEB crosslinking protocol and previously described in the NuXL rescoring study was reanalyzed and provided as example data (Siraj et al., 2024). Detailed information on sample preparation, experimental conditions, and mass spectrometric acquisition is provided in the original publication, and the raw data are publicly available via the ProteomeXchange repository under accession PXD048145.

Initial crosslink identification was performed using the NuXL search engine. The NuXL configuration specified a maximum peptide length of 30, a minimum peptide length of 5, an oligonucleotide length of 2, and a preset setting of RNA‐DEB. The reference proteome for *Escherichia coli* (canonical sequences, 4400 entries) was downloaded from the UniProt repository on 10.03.2022 and supplemented with the MaxQuant contaminant database (version 2.0.3.0) and used as the search database. The resulting FASTA file is provided in NuXLApp together with the example dataset. All other parameters were set to the NuXL default values. Subsequently, intensity‐ and retention time‐based data‐driven features were calculated within the Rescoring pipeline and combined with the NuXL default search‐engine features. These features were then used by Percolator to rescore the identifications. For DIA library generation, we used the same example file together with the crosslink and peptide output files generated by the NuXL search engine. All analysis steps, including executing the NuXL search engine with specified parameters, rescoring with incorporated data‐driven features, generating the DIA library, and manipulating results, were performed within a single NuXLApp workspace.

## 3 RESULTS AND DISCUSSION

NuXLApp provides accessible and intuitive data processing for protein‐Nu XL‐MS data analysis, combining data input, execution of the NuXL search engine, rescoring with data‐driven features, DIA library generation, and interactive result interpretation within a single application. To demonstrate the applicability of the complete NuXLApp workflow (Figure 2), the pipeline was applied to a representative example crosslinking dataset taken from a fraction of enriched peptide‐RNA oligonucleotide crosslinks derived from *E. coli* treated with 1,2:3,4‐diepoxybutane (Siraj et al., 2024). The workflow was executed without external preprocessing and generated all intermediate and final outputs within a single workspace.

First, we reanalyzed the provided example file of enriched peptide‐RNA oligonucleotide crosslinks using the NuXL search engine with the “RNA‐DEB” preset (Siraj et al., 2024). The NuXL search engine identified 972 CSMs at 1% CSM‐level FDR. Analysis of amino acid‐specific crosslinking efficiency at 1% CSM‐level FDR revealed that methionine (M), cysteine (C), and histidine (H) were the residues most frequently involved in crosslinking among all identified CSMs (Figure S6). In addition, the crosslink modification summary showed that the G + C4H6O2 adduct was identified most frequently, followed by A + C4H6O2 and AG + C4H6O2 (Figure S7). Subsequently, intensity‐ and retention time‐based data‐driven features were incorporated through the rescoring pipeline, combined with the default search engine features, and rescored by Percolator. Rescoring with additional data‐driven features increased the number of identified crosslinks to 1054 CSMs (+8.4%) at the same 1% CSM‐level FDR (Figure 3). The spectral library generation for DIA was carried out without iRT alignment. A total of 972 crosslink entries and 505 peptide entries were initially loaded. Following decoy filtering and deduplication, 530 crosslinks and 270 peptides were retained for library construction, yielding a final spectral library with 775 unique precursors, 306 proteins, and 18,508 fragment ion entries.

**FIGURE 3:**
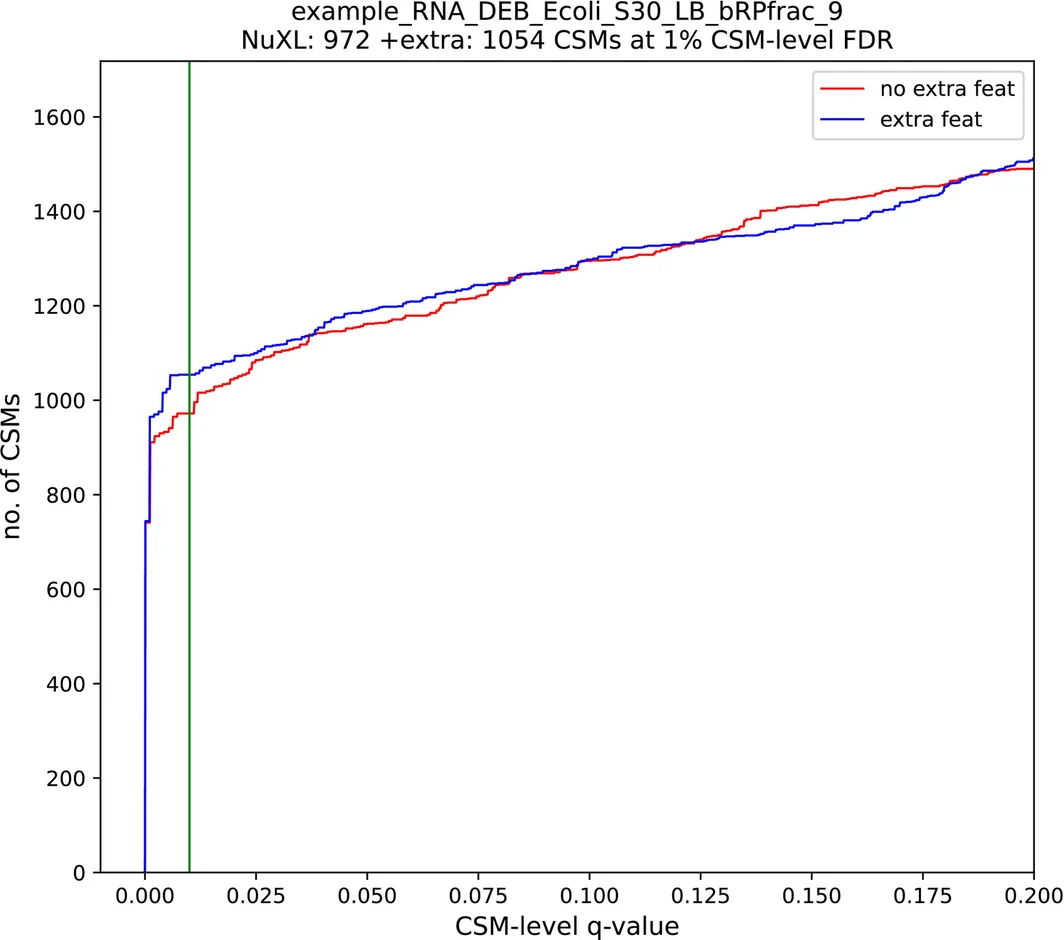
Effect of data‐driven features on CSM rescoring. The curves show the cumulative numbers of accepted CSMs across CSM‐level q‐value thresholds using the original NuXL feature set (no extra feat) or the NuXL features supplemented with retention‐time‐ and fragment‐intensity‐based features (extra feat). At a CSM‐level q‐value threshold of 0.01, the analyses using the original and supplemented feature sets yielded 972 and 1054 CSMs, respectively. The vertical line indicates the 1% CSM‐level FDR threshold.

NuXLApp integrates the NuXL search engine, rescoring, library generation, and visualization in a single interface. During installation on Windows, NuXLApp deploys the required OpenMS executables, DLLs, and related components needed to run the NuXL search engine. Thus, users do not need to install or configure OpenMS separately, nor manually assemble a Python environment for downstream analysis. This lowers the setup cost for users without a bioinformatics background. As a modular implementation of the OpenMS WebApps framework, NuXLApp can be readily extended with additional modules for downstream analysis (e.g., Rescoring and DIA library generation implemented in this study). NuXLApp uses MS2PIP for fragment ion intensity prediction, with the current implementation focusing on conserved fragment ion intensities. Training or fine‐tuning prediction models for crosslinked ions based on verified CSMs represents an important direction for future development. Because of the modular design of NuXLApp, alternative or fine‐tuned intensity prediction models can be incorporated into future versions, allowing users to select the most appropriate model for their data and analysis workflow. The modular page structure lets developers extend the app or use it as a template for new OpenMS WebApps. NuXLApp integrates an interactive spectra viewer into the workflow for validation of crosslinked spectra. Once the analysis is complete, users can inspect crosslink‐annotated spectra within the same interface, without the need to upload external files or switch to another application. In addition to spectra visualization, NuXLApp provides direct access to all key result components. Users can interactively explore the CSM table, protein (PRTs) table, and PRTs summary, as well as graphical outputs such as crosslink efficiency plots and precursor adduct summaries. All visualizations are interactive and can be downloaded in various formats.

NuXLApp eliminates the need for external tools, ensures that all relevant results are accessible in one place, and facilitates a seamless transition from identification to interpretation in protein‐Nu XL‐MS workflows. Together, these features lower the barrier to comprehensive analysis and interpretation of protein‐Nu XL‐MS data. NuXLApp was developed based on the OpenMS WebApps framework, which provides a workspace‐based architecture that supports reproducible analysis. These workspaces can be bookmarked and shared with other users to support collaboration. Beyond the hosted web application, NuXLApp is also available as a Windows executable. In addition, the platform can be deployed within local organizational infrastructure with Docker and Docker Compose files, which provide support for online hosting.

NuXLApp is deployed as a publicly accessible web service, and its complete source code is openly available, allowing users to deploy the application on their own infrastructure if desired. The hosted service runs on the de.NBI cloud and can scale across multiple compute nodes. In addition, a local Windows version is provided that offers the same web interface while executing all analyses locally. For the hosted service, computational jobs are managed through a queue to prevent server overload. Large submissions, including studies comprising hundreds of mzML files, can therefore be queued and processed as computational resources become available rather than requiring all files to be processed simultaneously. Once a job has been submitted, users can close the browser and return to their workspace later to inspect the results. Online workspaces are automatically deleted after 2 weeks of inactivity. The Windows version processes files sequentially and can likewise be used for large studies. Local processing typically requires approximately 8 GB RAM and can therefore be performed comfortably on a modern laptop. Unlike the hosted service, local workspaces are retained indefinitely and can be renamed and organized by the user.

## AUTHOR CONTRIBUTIONS

**Arslan Siraj:** Conceptualization; software; writing – original draft; methodology; investigation; formal analysis; validation; visualization. **Sergei Moshkovskii:** Conceptualization; investigation; formal analysis; writing – review and editing; methodology. **Tom David Müller:** Software; writing – review and editing. **Henning Urlaub:** Supervision; funding acquisition; resources; writing – review and editing. **Oliver Kohlbacher:** Writing – review and editing; resources; supervision; funding acquisition. **Timo Sachsenberg:** Conceptualization; software; methodology; writing – review and editing; supervision.

## CONFLICT OF INTEREST STATEMENT

Oliver Kohlbacher, Tom David Müller, and Timo Sachsenberg are officers in OpenMS Inc., a nonprofit foundation that manages the international coordination of OpenMS development. All remaining authors declare no competing interests.

## Supporting information


**Figure S1.** NuXL search configuration and workflow execution in NuXLApp. The workflow is organized into three sections: Files, Configure, and Run. In the Files section, users synchronize and select MS data and FASTA databases available in the workspace. The Configure section allows users to define the NuXL search parameters, including fixed and variable modifications with their corresponding mass shifts. In the Run section, the workflow can be started or stopped, while its progress is displayed in a live execution log that refreshes automatically. After completion, the generated NuXL identification files are stored in the workspace and can be downloaded directly as a bundled output archive or accessed through the results interface.
**Figure S2.** Rescoring with data‐driven features in NuXLApp. NuXLApp enables the incorporation of data‐driven features derived from the machine‐learning models described in our previous work (Siraj et al., 2024; Altstein, 2015). The workflow is organized into Files, Configure, and Run sections. In the Files section, users can synchronize existing workspace files or upload an initial NuXL identification file directly; the corresponding idXML and MGF files are then displayed for selection. In the Configure section, users select the identification file, the associated MGF file, and the appropriate crosslinking protocol, and can independently enable retention‐time prediction features, maximum‐correlation features, and generation of a pseudo‐ROC curve. Retention‐time features are calculated using fine‐tuned models for the selected crosslinking protocol, whereas the maximum‐correlation feature represents the highest correlation between the first three predicted prefix and suffix ion series obtained from the MS2PIP intensity model. The selected features are appended to the NuXL search engine and used for Percolator‐based rescoring. In the Run section, users can initiate the workflow and monitor its progress through the execution log. After completion, a CSM‐level pseudo‐ROC curve compares rescoring with the additional data‐driven features against rescoring without these features. The rescored identification file, pseudo‐ROC curve, and associated output files are saved to the workspace and can be downloaded as a bundled archive.
**Figure S3.** DIA spectral library generation workflow in NuXLApp. The workflow is organized into Files, Configure, and Run sections. In the Files section, users synchronize the available MS and idXML files from the workspace and may optionally upload an MSFragger‐derived TSV library for indexed retention time (iRT) alignment. In the Configure section, users select one or more mzML files, specify an output file‐name tag, choose the iRT calibration model, and determine whether mzML FileInfo processing and RDDF‐filtered identifications should be used. The corresponding NuXL identification files are automatically matched to the selected MS files. When an MSFragger TSV library is provided, it is used to calibrate retention times before library construction; otherwise, library generation proceeds without iRT alignment. The workflow converts NuXL DDA search results into a DIA‐NN‐compatible spectral library. In the Run section, users initiate the workflow and monitor its progress through the execution log. After completion, the generated library and associated output files are saved to the workspace and can be downloaded as a bundled archive.
**Figure S4.** CSM table and interactive visualization of annotated crosslinked spectra in NuXLApp. Users can select a NuXL identification file and inspect the corresponding crosslink‐spectrum matches (CSMs) in an interactive table with table‐download options. Selecting a CSM displays its annotated MS/MS spectrum below the table. The displayed annotations can be restricted to all matched fragments, b ions, y ions, b/y ions, non‐b/y ions, nucleic acid‐modified fragment ions, or precursor ions, thereby reducing spectral complexity and facilitating interpretation of individual CSM.
**Figure S5.** Proteins table and summary in the NuXLApp. The PRTs table provides crosslink information at the amino acid level, detailing UniProt accession, amino acid position, crosslink modifications, CSMs, and precursor counts, categorized into unique and shared identifications. The PRTs summary provides protein‐level details, and CSM counts for unique and shared peptides per protein.
**Figure S6.** Crosslinking efficiency output in the NuXLApp. The bar chart displays the normalized frequency of amino acids involved in crosslinks at 1% CSM‐level FDR. In this example, methionine (M) and cysteine (C) exhibit the highest crosslinking efficiencies, followed by histidine (H), while other residues show lower crosslinking frequencies.
**Figure S7.** Summary of crosslinked precursor adducts in NuXLApp. The pie chart shows the relative distribution of identified precursor modifications in the analyzed dataset at 1% CSM‐level FDR.

## Data Availability

The source code of NuXLApp is publicly available on GitHub (https://github.com/Arslan-Siraj/nuxl-app) under the permissive BSD 3‐Clause license. Deployment workflows are maintained in the OpenMS Streamlit deployment repository (https://github.com/OpenMS/streamlit-deployment). The integrated NuXL rescoring and spectral library generation tools are available at https://github.com/Arslan-Siraj/NuXL_rescore and https://github.com/timosachsenberg/NuXLDIA, respectively.
