# Supplementary material for: NuXLApp: Interactive data analysis and visualization for protein‐nucleic acid crosslinking mass spectrometry
Source: Protein Sci. 2026 Oct 15;35(11):e70820. doi: 10.1002/pro.70820 (PMC13673131; doi:10.1002/pro.70820)
Supplement: Supplementary file 1 [file PRO-35-e70820-s001.docx]

# **Supporting Information**

# **NuXLApp: Interactive Data Analysis and Visualization for Protein-nucleic acid crosslinking mass spectrometry**

Arslan Siraj^1,2, †^, Sergei Moshkovskii^3,4, †^, Tom David Müller^1,2^, Henning Urlaub^3,4^, Oliver Kohlbacher^1,2,5^, Timo Sachsenberg^1,2, *^

† These authors contributed equally to this work.

* Corresponding author: [timo.sachsenberg@uni-tuebingen.de](mailto://timo.sachsenberg@uni-tuebingen.de)

1. Applied Bioinformatics, Department of Computer Science, University of Tübingen, Tübingen, Germany
2. Institute for Bioinformatics and Medical Informatics, University of Tübingen, Tübingen, Germany
3. Department of Clinical Chemistry, University Medical Center Göttingen, Göttingen, Germany
4. Research Group Bioanalytical Mass Spectrometry, Max Planck Institute for Multidisciplinary Sciences, Göttingen, Germany
5. Translational Bioinformatics, University Hospital Tübingen, Tübingen, Germany

**Figure S1. NuXL search configuration and workflow execution in NuXLApp.** The workflow is organized into three sections: **Files**, **Configure**, and **Run**. In the Files section, users synchronize and select MS data and FASTA databases available in the workspace. The Configure section allows users to define the NuXL search parameters, including fixed and variable modifications with their corresponding mass shifts. In the Run section, the workflow can be started or stopped, while its progress is displayed in a live execution log that refreshes automatically. After completion, the generated NuXL identification files are stored in the workspace and can be downloaded directly as a bundled output archive or accessed through the results interface.

**Figure S2. Rescoring with data-driven features in NuXLApp.** NuXLApp enables the incorporation of data-driven features derived from the machine-learning models described in our previous work [1]. The workflow is organized into **Files**, **Configure**, and **Run** sections. In the Files section, users can synchronize existing workspace files or upload an initial NuXL identification file directly; the corresponding idXML and MGF files are then displayed for selection. In the Configure section, users select the identification file, the associated MGF file, and the appropriate crosslinking protocol, and can independently enable retention-time prediction features, maximum-correlation features, and generation of a pseudo-ROC curve. Retention-time features are calculated using fine-tuned models for the selected crosslinking protocol, whereas the maximum-correlation feature represents the highest correlation between the first three predicted prefix and suffix ion series obtained from the MS2PIP intensity model. The selected features are appended to the NuXL search engine and used for Percolator-based rescoring. In the Run section, users can initiate the workflow and monitor its progress through the execution log. After completion, a CSM-level pseudo-ROC curve compares rescoring with the additional data-driven features against rescoring without these features. The rescored identification file, pseudo-ROC curve, and associated output files are saved to the workspace and can be downloaded as a bundled archive.

**Figure S3. DIA spectral library generation workflow in NuXLApp.** The workflow is organized into **Files**, **Configure**, and **Run** sections. In the Files section, users synchronize the available MS and idXML files from the workspace and may optionally upload an MSFragger-derived TSV library for indexed retention time (iRT) alignment. In the Configure section, users select one or more mzML files, specify an output file-name tag, choose the iRT calibration model, and determine whether mzML FileInfo processing and RDDF-filtered identifications should be used. The corresponding NuXL identification files are automatically matched to the selected MS files. When an MSFragger TSV library is provided, it is used to calibrate retention times before library construction; otherwise, library generation proceeds without iRT alignment. The workflow converts NuXL DDA search results into a DIA-NN-compatible spectral library. In the Run section, users initiate the workflow and monitor its progress through the execution log. After completion, the generated library and associated output files are saved to the workspace and can be downloaded as a bundled archive.

**Figure S4. CSM table and interactive visualization of annotated crosslinked spectra in NuXLApp.** Users can select a NuXL identification file and inspect the corresponding crosslink-spectrum matches (CSMs) in an interactive table with table-download options. Selecting a CSM displays its annotated MS/MS spectrum below the table. The displayed annotations can be restricted to all matched fragments, b ions, y ions, b/y ions, non-b/y ions, nucleic acid-modified fragment ions, or precursor ions, thereby reducing spectral complexity and facilitating interpretation of individual CSM.

**Figure S5: Proteins table and summary in the NuXLApp.** The PRTs table provides crosslink information at the amino acid level, detailing UniProt accession, amino acid position, crosslink modifications, CSMs, and precursor counts, categorized into unique and shared identifications. The PRTs Summary provides protein-level details, and CSM counts for unique and shared peptides per protein.

**Figure S6: Crosslinking efficiency output in the NuXLApp.** The bar chart displays the normalized frequency of amino acids involved in crosslinks at 1% CSM-level FDR. In this example, methionine (M) and cysteine (C) exhibit the highest crosslinking efficiencies, followed by histidine (H), while other residues show lower crosslinking frequencies.

**Figure S7: Summary of crosslinked precursor adducts in NuXLApp.** The pie chart shows the relative distribution of identified precursor modifications in the analyzed dataset at 1% CSM-level FDR.

**Supplementary References:**

1. Siraj, A., Bouwmeester, R., Declercq, A., Welp, L., Chernev, A., Wulf, A., ... & Sachsenberg, T. (2024). Intensity and retention time prediction improves the rescoring of protein‐nucleic acid cross‐links. Proteomics, 24(8), 2300144.
